# Lead Poisoning and Intelligence: A Search for Cause and Effect in the Scottish Mental Surveys

**DOI:** 10.1155/2019/8980604

**Published:** 2019-11-12

**Authors:** Conrad Krebs

**Affiliations:** The LEAD Group Inc., Summer Hill, NSW 2130, Australia

## Abstract

In 1932 and again in 1947, the Scottish Council for Research in Education conducted the Scottish Mental Surveys. Testing two cohorts, one in 1932 and another in 1947, researchers set out to measure—using the same validated test each time—the intelligence of every Scottish child 11 years of age. The stated impetus for the Surveys was a fear that average Scottish intelligence was declining. But when investigators compared the results of the 1947 Survey with those from 1932 their predictions were completely upended. Instead of average intelligence declining, it had risen, substantially. The author argues that based on a study of the relevant ecosystems in place in Scotland at the time the increase in intelligence resulted from a decline in lead body burden. There is no evidence that the children were tested for lead. The decline is thought to have closely followed a fall in occupational lead use, a heightened awareness of the dangers of lead-solvency, improvements in lead plumbing in working-class homes, and a national campaign to improve the nutrition of women and children. Evidence shows that milk consumption in Scotland increased sharply, especially among children, beginning in the mid-1930s, just prior to and following the birth of the second cohort. This provided a source of calcium in a diet that had shown signs of deficiency. Evidence also suggests that lead contamination, from lead water pipes and industrial sources, was widely prevalent in Scotland in the early part of the twentieth century.

## 1. The Surveys

In 1932 and again in 1947, the Scottish Council for Research in Education, at the urging of the Population Investigation Committee and the Eugenics Society, undertook the Scottish Mental Surveys [1]. Testing two cohorts, one of 87,498 children in 1932 and another of 70,805 children in 1947, researchers set out to measure—using the same validated test each time (*see note 14*)—the intelligence of every Scottish child 11 years of age (*see notes 1 and 5 and p. 85 in [2]*). The stated impetus for the Surveys was a fear that average Scottish intelligence was declining, perhaps due to a tendency for the more intelligent members of society to produce fewer progeny than those who were less intelligent [1, 3]. But when investigators compared the results of the 1947 Survey with those from 1932 their predictions were completely upended. Instead of average intelligence declining, as was feared, it had risen, substantially [2].

With the 1947 results in hand, investigators began to look at cause and effect. Although they felt that environmental influences had undoubtedly played a role there has been no agreement on cause [1]. In a retrospective, Deary and co-authors believed that the 1947 results were an early example of a phenomenon famously described by psychologist James Flynn [4]. Flynn observed that when the same intelligence test was administered to successive generations of test takers, the current generation invariably scored better on the test than did previous generations regardless of test design [5]. Flynn theorised that intelligence increases in response to an increasingly complex world [6].

If Flynn's theory explains why children in 1947 were more intelligent than those in 1932 then the story ends and the Scottish Surveys return to obscurity. But there is an alternate theory, one that comes from studying children who have lived in leaded environments. As applied to the Scotland of the early twentieth century, the theory holds that as environmental lead contamination declined children in the 1947 Cohort absorbed less of the metal. It will be argued that together with changes in the social ecology of the country the decline resulted in a reduction in lead body burden. This reduction it is believed allowed children in the 1947 Cohort to perform better on the intelligence test than children in the earlier cohort.

## 2. Lead in Drinking Water

“*Roughly 150 years ago, cities all over the world installed lead pipes to distribute water,*” wrote Dr. Werner Troesken in his book *The Great Lead Water Pipe Disaster* [7]. Municipally owned lead service pipes for the delivery of drinking water existed in Scotland until well into the early part of this century [8]. In the mid-1970s, in response to growing concerns about lead in the environment, authorities conducted the most comprehensive survey of lead in water ever undertaken in Great Britain [9]. What they found was that the amount of lead in drinking water correlated directly with the lead content of the pipes and fixtures supplying the water [9]. As part of the survey, authorities drew water samples from 2,317 randomly selected households in England, 574 in Scotland, and 290 in Wales [9]. After confirming that the survey's demographics matched those of the countries under study, investigators extrapolated their findings. Based on “first draw” samples (*see note 6*) they estimated that 45.8% of homes in England, 58.4% in Scotland, and 37.9% in Wales had lead levels above a detectable minimum [9]. For the highest detected levels of lead (0.3 mg/L and greater), there was an even greater difference; 0.6% of homes in England, 11.3% in Scotland, and 0.8% in Wales were estimated to have such levels [9]. For “daytime” samples (*see note 7*) the differences trended in the same direction albeit with different numbers [9]. Lead-solvency, here defined as any process able to release lead into drinking water whether corrosion is present or not, explained much of the difference.

### 2.1. Lead Content of Household Plumbing

In the presence of lead plumbing the amount of lead in drinking water may be increased by: (a) increase in water acidity, (b) increase in water temperature, (c) age of the plumbing, (d) the use of lead-lined residential storage tanks, (e) the use of pipes made of different metals (usually copper and lead), (f) contact time between lead and water, (g) water pressure and turbulence of flow and, (h) thermal expansion of the pipe [10]. It can also be increased through the disruption of pipe deposits from public water flow cycling on and off and, as described above, the total lead content of the pipes and fixtures that connect the water main—which was usually made of steel or iron, not lead—with the household taps [11, 12]. In Scotland acidic water was the major reason for lead-solvency.

Reliable “always flowing” public water systems were uncommon in Great Britain until well into the twentieth century. For a variety of reasons, water service was routinely interrupted for several hours each day forcing the householder to look for other ways to keep water available at the tap [13, 14]. The usual solution was the household “cistern,” a large tank integrated into the plumbing that held as much as several hundred gallons or more. Frequently this was a large box made either of wood lined with lead or wholly of lead [12]. Cisterns were also common in rural areas where household water needs were met solely through the collection and storage of rain [14]. Although persistent published reports appeared alerting readers to the risk of poisoning from lead cisterns, improvements to plumbing infrastructure were not quickly or widely adopted. When they began to be implemented as part of new housing construction, it was too little and too late for the 1932 Cohort, all of whom had been born in 1921.

### 2.2. Obstacles to Plumbing Improvements

Although there had long been a recommendation to construct cisterns and pipes of lead-free materials in lead-solvent areas, there was a countervailing belief that given the right water chemistry further dissolution and corrosion of plumbing would be prevented once the action of water on lead had laid down a protective and impermeable barrier of metallic salts, which combined with organic matter in the water would shield the soft bare metal from further damage [11, 14, 15]. The problem would correct itself in effect. However the barrier could become damaged for any number of reasons, exposing the consumer to lead once again. A second obstacle to the adoption of plumbing improvements was that lead plumbing, particularly lead cisterns, were practically indestructible, as one observer noted, leading to extraordinarily long replacement cycles. In an investigation of a spontaneous outbreak of lead poisoning in the Scottish Highlands in 1974, the source of the lead was found to be the plumbing and in particular the lead cistern, which in one case was almost 200 years old [16]. Finally, in the first half of the twentieth century the vast majority of Scotlanders did not own the homes they lived in. They rented. It is estimated that in 1914 this circumstance applied to 90% or more of the population of Scotland [17]. That meant that the average householder did not have the legal right to remove lead from the plumbing even if he or she wanted to, and in the absence of government mandates or financial penalties, the landlord had no incentive. Improvements would have to wait for government action on housing as well as official recognition that lead poisoning from drinking water was a problem.

### 2.3. New Housing and Plumbing Improvements

In an official report published in 1917 on the housing problem attendant to the working classes of Scotland, investigators concluded that 236,000 new houses needed to be constructed throughout the country, both to replace houses unfit for habitation and to raise overall living standards to an acceptable level [18]. Beginning in 1919, as a result of the Commission's findings, a series of government building programs were initiated. By 1923 roughly 25,000 of the 236,000 houses had been built [19]. From 1923 onward new working class houses went up at the rate of about 7% of projected total each year. By the end of 1936 some 273,000 houses had been completed, 37,000 more than had been originally planned [20–29].

As households were moved out of old quarters and into new, trading old lead plumbing for new in the process, the risk of lead poisoning from drinking water dropped fractionally. Although this was particularly true where drinking water was not supplied from a lead cistern in the new house, simply trading old lead pipes for new lessened the risk of lead-solvency at least for a time. A review of published sources suggests that over the life span of a lead water pipe, which was said to average 50 + years, lead-solvency progressed through three stages: (1) the “conditioning” stage—in this stage lead-solvency began high and then gradually diminished as a protective barrier was laid down on the interior surface of the pipe. This stage was present whenever new or repaired lead pipe was placed into service. (2) The “conditioned pipe” stage—in this stage lead-solvency and corrosion were at their lowest. The length of this stage was determined by the adherence and permeability of the protective barrier. (3) The “aged lead plumbing” stage—in this stage both corrosion and lead-solvency increased as a result of failure of the protective barrier. “Aged lead plumbing” was found to be a reliable predictor of lead-solvency [10, 30]. The importance of the stages within the context of the Surveys is that during the children's most formative years, a greater number in the earlier Cohort grew up with plumbing in stage 3 (where the average age of working class housing was older), whereas in the 1947 Cohort a greater number grew up with plumbing in stages 1 and 2 (where the average age of working class housing was newer; *see note 13*).

How often builders failed to include a lead cistern in new construction is not known. However in the 1980s 500 children living in central Edinburgh were surveyed as part of the Edinburgh Lead Study [31]. At the time it was estimated that there were between 30,000 and 60,000 lead-lined water storage tanks still in use “for all domestic purposes” mainly in the Edinburgh and Glasgow areas [10]. In the follow-up study investigators examined the cold water supply in 480 of the children's homes. Non-public housing that was rented as opposed to owner occupied was more than twice as likely to have cold water supplied from a lead storage tank. The difference between public housing and privately rented homes was even starker, the latter being 10 times more likely than the former to have cold water supplied from a lead tank (see ref. p. 197 in [31]). The study also found that more recently built homes were far less likely to include such tanks than were houses built during the late Victorian period or early twentieth century [31]. In 1931, in an obscure reference came a hint that municipal authorities were ready to acknowledge that lead-solvency was a problem [32] (*see note 15*). And in 1938, 2 years after the birth of the 1947 Cohort, lead-solvency made an appearance before the law. As reported in a lecture before the Royal Institute of Chemistry of Great Britain and Ireland on water and public health, the speaker stated that a judge had ruled that water companies had a duty to either warn customers of the danger of lead-solvency or take steps to mitigate it [33].

## 3. Occupational Lead: Another Source of Childhood Lead Exposure

### 3.1. The Shipbuilding Industry

Spanning the width of Scotland from the Firth of Forth on the east coast to the Firth of Clyde on the west lay the heart of the country's industrial might for much of the nineteenth and early twentieth centuries. And at the centre of that lay the largest shipbuilding industry the world had ever known. Shipyards were dirty, dangerous places [34], and often hidden in the dirt was dust and residue from lead. Lead was widely used in shipbuilding. It could be found in paint and plumbing (as pipe, conduit, and solder), as a lubricant, in engine parts, as corrosion control coatings on plate steel, and as sheet lead for floors and bulkheads in areas subject to condensation such as galleys and room-sized refrigerators [35]. The dangers from occupational lead exposure were never greater than during shipbreaking, the dirtiest most hazardous work that a shipyard could engage in. Shipbreaking surged at the end of the Great War, a result of obsolete naval vessels being scrapped [36]. In 1924 the number of cases of lead poisoning reported among workers in the British shipbreaking industry was higher than 15 other lead-related occupations and industries and 30% higher than the next highest number [37].

After peaking in 1920, shipbuilding in Scotland went into decline and never fully recovered. Although the industry rebounded somewhat in the late '30s, gains in worker productivity as well as a migration of skilled labour away from the industry during its lean years reduced the insured work force from 97,000 in 1921 to 41,000 in 1936 (*see note 3*) [38]. That meant that far fewer wage earners were potentially bringing lead-contaminated clothing home to be laundered. That change would benefit children in the 1947 Cohort, roughly 80% of whom had working class parents (*see note 2 and p. 215 in [1]*).

### 3.2. The British Admiralty and Occupational Lead Safety in Shipyards

By 1920 what promised to be difficult years ahead for the Industry became even more so with the withdrawal of the British Admiralty from the business of building ships, a move required by international treaty [39, 40]. In addition to removing a critical source of industry funding, the withdrawal removed a role model for lead safety [39, 41].

“*The Admiralty have for some time recognized the dangers attaching to the use of lead paints, and have taken precautions accordingly*.” That statement, given in testimony before a committee investigating the dangers of lead paint, revealed how seriously the British Navy took the threat of lead poisoning among ship's painters. The point was driven home by the fact that the Navy had become a leader in the use of lead-free paints on the interior surfaces of ships [42]. A man painting ships for the Navy could expect to benefit from the following, all provided at Admiralty expense: plenty of soap, nail brushes, hot water, and towels and the time to use them before lunch and at shift's end; enforcement of the preceding by an overseer stationed at the door of the washroom (“no wash-up” meant “no wages”); work overalls and their regular washing at a commercial laundry, a benefit often denied those working on civilian ships; and regular examinations for signs of poisoning [42, 43]. Although the speaker was referring to activity at Government-owned dockyards, it is illogical to believe that the Admiralty would abandon this or any regulation simply because a naval vessel was being built, refitted, or repaired at a commercial rather than at a navy yard.

Admiralty contracts contributed roughly five times that of merchant contracts to yard overhead and profit at two of the leading Clyde shipyards in the years leading up to World War II [39]. That kind of financial security would have made the Admiralty the most important customer for any yard lucky enough to have the Navy's business. So, it is difficult to imagine a scene where ship's painters are up to their elbows in soapy water before mealtime at a berth where a navy vessel is being built, while at an adjacent berth in the same yard, workers painting a merchant ship are sitting down to eat with paint-encrusted hands. Although that may have occurred, a more optimistic view is that the Admiralty's position on lead safety would have been embraced by yard management. But, as the shipbuilding industry fell into disarray from 1920 onward, prompting yard management to cut costs, the painters, never well organised to begin with, lost ground. An example was the demise of an agreement reached at the end of the Great War between labour unions and yard management for the latter to provide clean overalls on a regular basis. That benefit would disappear sometime before 1930. As World War II approached, and commercial shipyards revived, painters regained much if not all of their lost ground [43], in step with the return of the Admiralty to shipbuilding.

### 3.3. The Munitions Industry

The other source of occupational lead was the munitions industry which in Scotland as elsewhere employed tens of thousands of women during the Great War (*see [45], note 4, and p. 200 in [44]*). In addition to the ever present risk of fire and explosion, the hazards of munitions manufacturing were made worse by the fact that many workers and managers, inexperienced and hired in a frenzy after production was found to have fallen far short of need, were unfamiliar not only with munitions work but with heavy industry of any kind [44]. That unfamiliarity likely exacerbated the risks of an acknowledged lead hazard.

In a munitions factory, lead could be found in paint to protect the finished product from corrosion, in brass shell cartridges to make them easier to machine, as an essential component of artillery Shrapnel shells, in soldering and tinning processes, and to harden and temper metal used in making bombs and shells. As part of the hardening process, the metal was often immersed in a bath of molten lead to bring the former to a uniform temperature [46, 47]. Evidence has shown that any time an object is immersed in a lead bath, the thin layer of lead oxide floating on the surface of the molten lead breaks into pieces, some microscopic in size. The smallest of the pieces may become airborne floating as invisible particles [48]. What precautions were taken to prevent these particles from being inhaled, or whether authorities were even aware of the problem, is unknown. However, by War's end, 12,000,000 artillery shells had been produced in Scotland alone (*see p. 22 in [44]*). Although not all shells and bombs required treatment, many if not most Shrapnel shells, a type used extensively by the British and in enormous quantity, were heat treated during manufacturing either by immersing them in molten lead or by other means.

Although the Ministry of Munitions' Committee on the Health of Munitions Workers issued comprehensive safety guidelines intended for the protection of workers, serious questions have been raised as to how closely these guidelines were followed in light of the relaxation of labour laws governing the conditions of industrial employment for the duration of the Great War (*see [47] and p. 177 in [44]*). Given lax enforcement, it would be difficult to believe that there were not more than a few women who emerged from the experience having absorbed lead at their place of work, a claim for which there is some evidence.

Sometime after the signing of the Armistice in 1918, the Ministry of Munitions' Committee on the Health of the Munition Workers issued its final report. Included in the report was a section written by a senior medical officer who in 1917 had supervised the medical examination of 1,183 women at eight munitions factories in England [49]. In 1915/1916, the same committee had arranged for the medical examination of 1,326 women at eleven munitions factories throughout Great Britain including Scotland (*see note 10 and p. 13 in [50]*).

In the two studies, in addition to other findings, up to 51% of women complained of constipation; up to 32% had several carious teeth; as many as 37% had complained of indigestion, abdominal pain, or loss of appetite; up to 30% of nervousness, irritability, depression, or difficulty sleeping; as many as 59% of recurrent headaches; and as many as 28% of muscular aches and pains (see p. 141 in [49] and p. 181 in [50]). In each study, the same complaints were noted to have come from workers at all factories although the frequency of each varied considerably from factory to factory [49, 50]. This suggests that the complaints were work related and not something that the women brought from home. All of the findings have been associated with proven cases of chronic lead poisoning. And while no single finding is specific for lead poisoning (each can be seen in other conditions), the fact that all of the findings were observed over a single time period, in a group of women all doing related work, with known occupational exposure to lead, and working in munitions plants that too often provided grossly inadequate washing accommodations for personal hygiene (even where lead was routinely handled) [50], makes it highly likely that lead was causative. The importance of this is that a number of these same women would go on to give birth two or three years later to children who would participate in the 1932 Cohort. That mothers can unknowingly poison their unborn child with lead, even years after maternal lead exposure has ended, is well documented [51, 52].

### 3.4. The Lead Paint (Protection against Poisoning) Act

In 1927 came the opening that would allow information on the dangers of lead to reach a wider audience. Issued in December of 1926 to go into effect January 1 of the following year, the Lead Paint (Protection Against Poisoning) Act made mandatory a host of new requirements for firms that used lead paint to paint buildings [53, 54]. The practical effect of the Act was to reduce the use of lead paint. “*The best that can be hoped,”* wrote Dr. E. L. Collis of the Act, “*is that employers, rather than be bothered with complying with the code, will adopt the use of paints which do not contain lead. There is already a considerable tendency in this direction”* [55]. Because the Act would have added to the cost of painting, official language on the dangers of lead paint would likely have appeared in every painting contract, every deal negotiated with a customer, and would have been conveyed to every employee retained, hired, or released by a firm. In this manner, public awareness of the dangers of lead had an opportunity to advance.

### 3.5. A Declining Incidence of Occupational Lead Poisoning

Occupational lead poisoning, a reportable disease in Great Britain for most of the twentieth century (although not until 1927 for building and carriage painters), fell in uneven fashion from a peak of more than 400 cases in 1923 (including almost 50 deaths) to 168 cases (including 17 deaths) in 1935 [37, 56, 57]. The decline occurred despite the fact that between 1931 and 1936 lead consumption increased by over 30% in Great Britain [35]. From the late '30s into the early '40s, the annual number of cases of occupational lead poisoning in Great Britain from all sources averaged around 115 with few if any deaths [58].

## 4. The Scottish Diet, Dietary Calcium, and the Absorption of Lead

“*The rapid advance in the science of nutrition in recent years has shown that the influence of diet on health and physique is profound*,” wrote John Boyd Orr, Scotland's leading nutritionist, in 1936, “*The results of research are now to some extent being applied*” [59]. A goal of Orr and others, in conjunction with this advance, was to increase children's consumption of milk, a goal that they had begun to realise, particularly in large urban areas, by the mid-1930s. “*Since … 1929 …*,” wrote Orr, “*… there has been much publicity pointing out the great value of milk as a food, particularly for children, and it is difficult to believe that this propaganda has been without effect on the dietary of the people*” [60]. In large part due to his influence, children in the 1947 Cohort enjoyed higher levels of dietary calcium than did children in the 1932 Cohort.

The importance of Orr's achievement, for present purposes, is that by increasing calcium intake, in this case by consuming milk, the absorption of lead from the gastrointestinal tract was reduced and the elimination of lead from the body increased [61]. Conversely, it has been shown that a deficiency of calcium in the diet will promote the absorption and retention of lead [61–64]. Beginning in late 1934, subsidised milk began to be given to children in Scottish schools on a daily basis, and by 1939, children ages 1 through 5 was well as expectant and nursing mothers were receiving milk as well. By the time the 1947 Cohort had reached age seven years (in 1943), 75% of the school age population in Scotland were receiving school milk daily [65]. There is evidence that calcium in the diet of working class Scots was deficient from before the Great War and throughout the 1920s (*see note 11*).

Writing in the journal *Environmental Health Perspectives* in 1979, Levander noted that studies in animals showed that ingestion of “hard” water, that is, water containing cations, including calcium, decreased the absorption of concomitantly ingested lead for the same reason milk does. Potable water in Scotland is acidic by reason of its “softness,” that is, it is water that does not contain acid-neutralising cations. “*People living in soft water areas appear to be in double jeopardy…*,” wrote Levander, “*since such water not only dissolves more lead from lead plumbing but also fails to provide any protection against absorption of lead by the gastrointestinal tract”* [64].

## 5. Questions Raised by the 1947 Survey

### 5.1. Gender Differences

Two of the more interesting questions raised by the 1947 Survey dealt with the participation of girls in the study and the distribution of test scores by educational regional authority, respectively. Of the former James Maxwell wrote that “*The question must remain open*, *one major difficulty, the fact that most of* the *increased score in 1947 was due to the girls, has to be accounted for in any explanation*” [3]. A proposal now put forward is that girls in the 1947 Cohort benefited more than boys from the decline in lead contamination of the home, a decline that began in the late 1920s.

In a Scottish working class home, the wife and daughters did the heaviest housework; washing clothes, scrubbing floors and staircases, and cooking. Boys had some domestic responsibilities as well, but more of their time was spent outside the house working at part-time jobs, the most common of which was delivering milk [66]. Old photos of the era show Scottish shipyard workers labouring in what appears to be street clothes, a conclusion supported by oral histories [34]. Pictures show vests, jackets, coats, trousers, and cloth caps being worn, occasionally supplemented by overalls. But unless the work was for the Admiralty, all the clothing including the overalls came home to be laundered (*see note 8*).

In houses that were home to more than one industrial worker, a not uncommon occurrence as many households sublet space for any one of several reasons [19], the quantity of contaminated clothing waiting to be washed could be increased many times over. Since laundry facilities for working class families were always communal, this made lead-contaminated clothing a hazard for others as well [17]. Lead poisoning in families from lead brought home from work as dust and residue had become such a problem in Great Britain that sometime around 1911, premiums for workmen's compensation insurance for painters were increased because of it [67]. But as events conspired to reduce occupational lead exposure—an unexpected benefit of the pullback of the shipbuilding industry, the return of the Admiralty to commercial shipyards, and the Lead Paint Act of 1927—the quantity of lead dust and residue in the home diminished, a clear benefit to children in the 1947 Cohort. “*It is interesting to note*,” wrote the authors of the 1947 results, “*that the superiority of the girls* [over the boys] *is most marked in the lower* [end of the range] *of score*” (see Table 1) [2].

Investigators identified a family's occupational class only in a randomly selected subset of Survey participants (The Thirty-Six Day Sample; *see note 12*). Within the subset, test scores that were roughly one standard deviation above, or below, the mean were labelled as either “high scorers” or “low scorers,” respectively. Ninety-four percent of children in the “low scorers” and 62% in the “high scorers” were from working class families (see p. 113 in [1]). If any improvement in test scores were to occur due to a reduction in occupational lead contamination of the home, it is in the “low scorers” that change would have been expected and was in fact seen.

### 5.2. Geographic Distribution of Test Scores

The second question concerns the distribution of test scores across the regional groups of educational authorities. A similar analysis was not completed for the 1932 Survey. “*The general trend*,” wrote James Maxwell, “*is a decrease in average score from the South-East to the West and North* [of Scotland] *with Orkney and Zetland* [islands in the far north of the country] *being exceptions*” [3].

An examination of topographical maps of Scotland reveals within two ecosystems a phenomenon that unexpectedly tracks the geographical distribution of test scores: the acidity of standing fresh water and the peaty soil that drains it [68, 69]. Moving from the south and east of the country (where average scores were higher) to the north and west (where average scores were lower) the acidity of both systems broadly increases (see figure, below; also see Harriman Figure 6.4, [68]).

**Figure 1:**
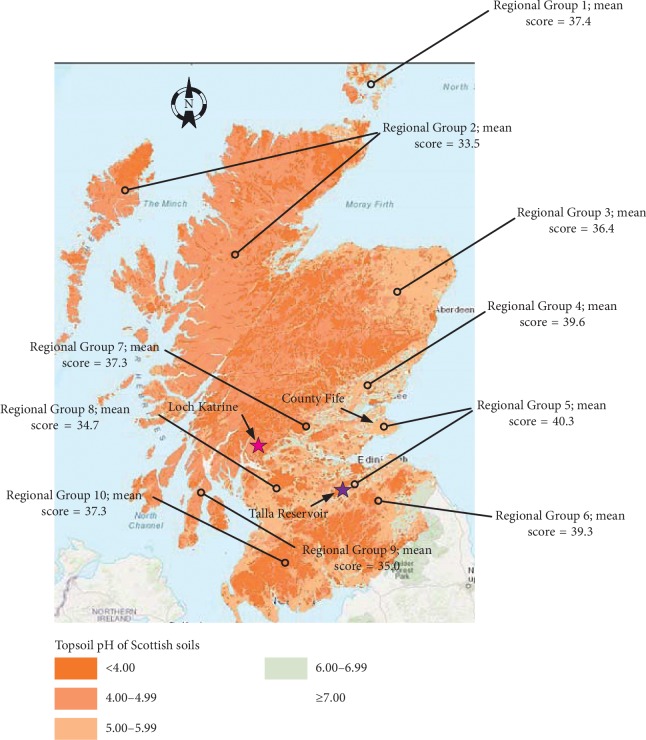
Map of median topsoil pH in Scotland^∗^. The location of each administrative region participating in the 1947 Survey is also shown. The map is constructed from data representing more than 40,000 analyses held within the Scottish soils database. Each open circle shows the approximate geographic center of the respective educational administrative region. Each region, which consisted of 2 or more pre-1974 Scottish counties, is numbered. Along with the number is given the mean Survey test score for the region. The two “star” symbols indicate the locations of the primary source of drinking water for Edinburgh (*Talla Reservoir*) and Glasgow (*Loch Katrine*) at the time of the Surveys (see text). Glasgow is part of group 8 while Edinburgh belongs to group 5 (^∗^contains James Hutton Institute material (copyright 2018) (http://ukso.org/SoilsOfScotland/home.html)).

In Figure 1 are also shown the main sources of drinking water for Glasgow (Loch Katrine) and Edinburgh (Talla Reservoir) at the time of the Surveys [70]. Talla lies within the River Tweed catchment, an area that for reasons of geology is more alkaline than that which surrounds Loch Katrine [68]. Throughout the country north northeast of Edinburgh and Glasgow (approximating Regional Groups 3 and 4 and the County Fife portion of Regional Group 5) standing fresh water generally retains the alkalinity found in waters south southeast of both cities. As one moves away from the two cities to either the northwest or far southwest of the country, areas where slow-weathering rock is more common, the acidity of standing fresh water comes to more closely resemble that of topsoil. As a result, both ecosystems show high levels of acidity in these locales [68]. From the map, Regional Group 10 might seem to be the exception to the rule that water acidity and mean test scores are inversely related. However the small urban centres of that sparsely populated area were clustered near the coastline close to areas of relative alkalinity [68]. In Orkney the fast-weathering nature of the geology there resulted in relatively alkaline fresh water despite the fact that the islands' drinking water came predominantly from lochs and reservoirs [71]. In Zetland drinking water likely came from wells, bore holes, and springs, not from surface sources given the sparse population of the region. This may explain why these two island groups were the exception.

The distribution of mean scores from the 1947 Survey is skewed by the fact that 62% of children in the Survey came from just 2 of the 10 educational administrative regions, regions 5 (which included Edinburgh) and 8 (which included Glasgow). If regions 5 and 8 are excluded from consideration, Maxwell's southeast to northwest trend subjectively remains although weakened considerably. The reverse is also true. When the other 8 regions are excluded and only regions 5 and 8 are considered, the trend persists. Together with Harriman's data, the figure suggests that an inverse relationship exists between drinking water acidity and mean test scores.

## 6. Discussion, Summary, and Conclusions

This is not the first time that the question of occult lead poisoning has arisen in connection with the intelligence of Scottish children. Writing in the journal *The Lancet* in 1975, Beattie et al. compared a group of mentally retarded children in Glasgow with a group of mentally normal children living in the same city. What they found was that “*the … difference between the two groups tested is the significant excess of retarded children coming from homes with high … lead concentrations in the water supply*.” The difference extended to blood-lead levels as well [72]. A number of children participating in the Surveys (less than 1% of either Cohort) continued to be studied for several years [73]. Although differences in educational attainment and rates of marriage and fertility were recorded, none of these have shed light on the difference in intelligence between the Cohorts.

In a seminal paper published in 1994, Dr. Stuart Pocock and colleagues systematically reviewed 26 epidemiologic studies in which inverse correlations between lead body burden and intelligence in children, aged 5 years or more, were found [74]. Despite concluding that low-level lead exposure may cause a small IQ deficit they cautioned that statistical uncertainty required that other explanations be considered as well [74]. Similar concerns have been raised by others [75, 76]. Although there is no proof that lead could be found in Scottish children in the early part of the twentieth century, the risk of lead contamination was at its height in Scotland at the time and public awareness of the dangers of lead was minimal. That a child living in a leaded environment is at very high risk of absorbing the metal is undisputed.

One of the more rigorous and well-designed studies in Pocock's review was that by Fergusson et al. [77]. Using a longitudinal design, the authors found no change in IQ following adjustment for covariates including gender. There remained however “*a weak* [and statistically significant inverse] *association between dentine lead levels and all measures of the child's school performance including word recognition and* [blinded] *teacher ratings of school achievement*” [77]. The authors discussed possible reasons why ability (that is, IQ) did not do a better job of predicting school achievement, including the possibility that the latter measure was more sensitive to lead burden. “*School performance measures*,” they wrote, “*reflects the child's acquisition of skills over a relatively long time period”* [77] unlike measures of the child's ability. In the 1947 Survey, among the 1,120 children randomly selected for formal IQ testing (the Six-Day Sample; children in the 1947 Cohort who were born on the first day of even months in the year 1936), there was evidence that girls did appreciably better on the verbal test than boys at a given IQ level. This was particularly true at the lower end of the test score distribution [2]. Like Fergusson then, this finding suggests that, under some circumstances, IQ does not predict academic performance as well as might be expected. Further evidence of this came in a longitudinal study of 70,000 healthy English school children, where girls consistently demonstrated superior academic performance at intelligence levels comparable to boys, an advantage that appeared to be constitutional [78]. This then raises the question as to whether the gains shown by girls in the Six-Day Sample were constitutional as well. However, that does not explain why girls trailed boys slightly in test performance in 1932 and then leaped ahead of them in 1947, by two orders of magnitude when the entire Cohort is taken into account (*see note 17*), or why their improvement was predominantly at the lower end of test score distribution. A difference between genders in lead body burden might explain both however. Since Fergusson and Pocock published their results, several others have reported data showing significant inverse relationships between lead body burden and intelligence [79–81].

Scottish investigators searched for an explanation for the increase in test scores, looking at genetic, sociologic, and social conditions in Scotland. They arrived at no conclusions [3]. They were also puzzled over gains made by the girls, speculating that “test sophistication” could have been the reason for improvement, but acknowledged that “*The difference* [between boys and girls in mean test score] *cannot be easily explained through any selective process and would appear to be environmental* [in origin], (see p. xiv in [2]).

Based on an interpretation of the evidence, and on the knowledge that lead can adversely affect a child's mental development, a theory is proposed to explain the rise in Scottish test scores. Following a fall in environmental lead levels, and in conjunction with higher intakes of dietary calcium, the theory states that the 1947 Cohort experienced lower lead burdens than did children in the 1932 Cohort (*see note 9*). As a result, children in the 1947 Cohort had an intelligence advantage over children in the earlier cohort and were able to perform better on intelligence tests. Causation argued from an ecological perspective, using evidence of social, environmental, and public health concerns in Scotland, does not prove or disprove that lead influenced the outcomes of the Surveys. To do that would have required further study of the children, an opportunity that unfortunately has been lost. The argument does suggest however a biologically plausible alternative to theories already proposed.

## 7. Notes

1. For the 1947 Survey, only children at school and in class the day of the Survey were counted. Children who otherwise satisfied study entry criteria but were absent from school that day (@ 4,500 children) are not included in the figures. No attempt was made to locate and test these children after the fact. It is not clear if either survey was announced in advance. If so, then some families may have elected not to allow their children to participate. No information is available on the number of children absent the day of the 1932 Survey.
2. From a random sample of 10% of the children (“The Thirty-Six Day Sample,” see also note 12), investigators in the 1947 Survey obtained information on family living conditions, father's occupation, the height and weight of the child, certain physical disabilities of the child, and other information. None of this information was obtained from children participating in the 1932 Survey.
3. In Great Britain, unemployment insurance was required of all workers in shipbuilding and allied industries. The total number of insured workers (employed and unemployed) was provided monthly in the *Ministry of Labour Gazette*. Casual employment was common in the shipbuilding industry. In any given month, a worker could work every day or only part of the month and often for different shipyards. In such a system, separating employed from unemployed becomes difficult. For that reason, the numbers 97,000 and 41,000 represent the total insured workforce which counts both employed and unemployed. From 1921 to 1936, the total insured workforce steadily declined.
4. Although Scott and Cunnison in their book *The Industries of the Clyde Valley During the War* cite a figure of 28,000 women employed in munitions in Scotland in October 1918, the authors acknowledged that the number does not include women from every munitions factory and workshop [45]. Myra Baillie has identified an additional 15,000 working in other munitions firms plus an additional 4 firms for which no figures are available (see p. 33 in [44]). Based on these and other sources, Baillie estimates that as many as 100,000 women were employed in all phases of munitions manufacturing and supporting industries throughout Scotland.
5. The intelligence test administered to children in either Cohort was not an IQ test [2]. In order to measure the IQs of all children in the Cohorts, samples of children were randomly selected from both Cohorts for formal IQ testing. After confirming that the demographics of the samples faithfully reflected the demographics of the Cohorts, intelligence test performance was then equated to a specific IQ for each child in both Cohorts.
6. A “first-draw” sample was water taken from the kitchen tap first thing in the morning before any other water in the house had been run. This is water that had sat in contact with lead all night.
7. A “day-time” sample was water taken from the kitchen tap at random times during the day before any water had been run off.
8. The topic of overalls was much discussed during the Committee on Lead Paint hearings of 1915. It was clear from testimony that some painters painted only in their street clothes and wore no overalls at all. Some employers testified that they would be willing to provide clean overalls for painters in their employ. Others would do so only under duress. In the end, the Committee recommended to the Home Secretary that painters be required to provide for the washing of their own overalls on a weekly basis [42]. When the law governing the use of lead paint in buildings was enacted in December of 1926, that recommendation was followed [53].
9. No relevant health information was collected from children in either Survey other than height and weight in a randomly selected subset of the 1947 Cohort (“The Thirty-Six Day Sample,” see also note 12). Those data showed that children with working class parents were generally shorter and lighter than children whose parents were professional, administrative, managerial, etc (*see p. 87 in [1]*). While the data are not diagnostic of chronic lead poisoning, neither are they inconsistent. Failure to thrive, a term used to describe children who fail to achieve their expected rate of growth and development, has been associated with chronic lead poisoning among other conditions [82].
10. The two studies are not comparable except to identify broad trends. Different teams of physicians were used to examine workers in each study. There is no evidence that cross-training of the two teams took place or that investigators made any effort to standardise examinations across studies. Although it was stated in the 1917 study that “every effort” was made to obtain results that were comparable across factories, there is no evidence that this was the case in the earlier study.
11. Dental caries were rampant in Scotland in the first quarter of the twentieth century, and rickets were common. Dietary calcium deficiency has been found capable of contributing to both conditions, and lead toxicity can contribute to dental losses independent of other causes [83–86]. In 1911, the Glasgow School Board reported that over 90% of school children had “bad teeth” and 50% had “very bad teeth” [87]. A 1939 dental examination of over 200,000 Glasgow school children found 56% with “one to four teeth decayed” while almost 30% had “sound teeth” [88]. Among school children in Scotland, the percentage with rickets was noted to have fallen from 9% in 1910 to 1.5% in 1937 [89].
12. Investigators found it impractical to determine socioeconomic status for every child in the 1947 Cohort. For children born on the first 3 days of each month in the year 1936 (“The Thirty-Six Day Sample”), they determined the occupation of the child's father along with other information. “*In each of the comparisons* [involving gender, test score, age, geographic distribution, and family size] *of the thirty-six-day sample with the whole eleven-year-old age group which has been made*,” wrote investigators, “*no difference between the sample and the population from which it was drawn has been statistically significant*” (see p. 10 in [1]).
13. Urban renewal of working class housing in Scotland—a program of new construction and slum clearance–gradually gained momentum beginning in the 1920s. Although only 11,000 condemned houses were razed in the ten years ending in 1929 [90], more than 70,000 houses, unfit for habitation, were leveled throughout the country between 1930 and 1938 [91]. Together with the completion of 273,000 new working class houses (in time for the birth of the 1947 Cohort, in 1936), the initiative came to dwarf the only previous one in Scotland which took place in Glasgow. There, between 1866 and 1914, approximately 16,000 condemned houses were leveled [92]. This roughly coincided with the construction of 90,000 new houses (of all types) in the city between 1870 and 1905 [93]. The larger scale of the second initiative, focusing as it did on working class housing, would come to benefit the 1947 Cohort in a way that the earlier Glasgow initiative would fail to benefit the 1932 Cohort (*see note 18*).
14. The verbal or Moray House Test, developed at the Moray House Training College, Edinburgh, Scotland ([4], p. 13).
15. The authors of this monograph, on the engineering challenges surrounding the use of lead alloys in building construction, acknowledged the problem of plumbosolvency (also known as lead solvency) in “domestic water service” and the importance of avoiding it by means of material testing ([32], p. 17). This was the earliest mention of plumbosolvency that the author was able to find in an official British government publication.
16. The average verbal test score increased from 34.5 in 1932 to 36.7 where the maximum was 76 points, a 2.9% increase (The Trend of Scottish Intelligence, p. viii).
17. The mean test score for all girls in the 1932 Survey trailed that of the boys by less than a tenth of a point. In the 1947 Survey, the girls' mean score led that of the boys by 1.8 points (see p. 85 in [2]).
18. There is no indication that urban renewal programs of any substance occurred in Scotland, outside of Glasgow, before 1920.

## Figures and Tables

**Table 1:** Distributions of high and low scorers by gender for both the 1932 and 1947 verbal tests as reported by the Survey's authors.

Distribution of verbal test scores for all high and low scorers, 1932 and 1947^*∗*^				
		Low scores (range 0–19)	High scores (range 60–76)	Total in cohort
Boys born in 1921	*N* (%)^+^	8672 (19.6)	1881 (4.3)	44,210
Girls born in 1921	*N* (%)^+^	7753 (17.9)	1428 (3.2)	43,288
Boys born in 1936	*N* (%)^+^	6773 (18.9)	2168 (6.0)	37,998
Girls born in 1936	*N* (%)^+^	4907 (14.0)	2098 (5.9)	37,213

The intelligence test used in both 1932 and 1947 was an instrument developed originally for the placement of students into secondary schools in England (Deary [4]). The test included a picture and verbal portion, the latter so called because “it required literacy and numeracy to understand and complete the items” (Deary [4]). The verbal test had a possible score of 76. ^*∗*^Adapted from data in Tables 6–9, pp. 83–84, in: *The Trend of Scottish Intelligence*; The University of London Press, 1949. Used by permission. ^+^ The percentage of high and low scorers in the total cohort, sorted by gender and year of birth, and their numbers.
